# How Do We Honor Identities in Medical Education? Commentary on Yang et al., 2025: Underrepresentation of Filipino, Laotian, Cambodian, and Indonesians Among US Allopathic Medical Students

**DOI:** 10.1007/s11606-025-10085-9

**Published:** 2025-12-18

**Authors:** Tonya L. Fancher, Jonathan Credo, Stephany Sanchez

**Affiliations:** 1 Department of Internal Medicine, Division of General Medicine, University of California, Davis School of Medicine, Sacramento, CA USA; 2 Department of Internal Medicine and Department of Psychiatry and Behavioral Sciences, University of California, Davis Health System, Sacramento, CA USA; 3 Department of Internal Medicine, Division of General Medicine, University of California, Davis School of Medicine, Sacramento, CA USA; 4 Department of Chemistry and Biochemistry, Northern Arizona University, Flagstaff, AZ 86011 USA

As the bicultural daughter (T.L.F.) of a Japanese immigrant who came to the US on the heels of the Korean War, I was raised to respect the diversity of Asian ethnicities. We were Japanese (others would say we were Hokkaidoan, as my mom was born on the island of Hokkaido), my mom’s best friend was Korean, and my brother’s girlfriend was Vietnamese. We were the only Asians among other people.

This issue’s paper by Yang et al.^1^ is a reminder of the workforce diversity strategy to fulfill medical education’s “social contract to achieve better health care for patients and better health in communities”.^2^ Numerous studies have found that a trusting relationship between caregiver and patient can strongly affect adherence and outcomes. While we can debate what constitutes a “good” doctor, for patients, an initial indicator of a potentially trusting relationship is homophily—the tendency for people to seek out those who are similar to themselves.

Yang and team used disaggregated data from the AAMC to compare male and female applicants and matriculants who self-identified as Bangladeshi, Cambodian, Chinese, Filipino, Indian, Indonesian, Japanese, Korean, Laotian, Pakistani, Taiwanese, Vietnamese, or other Asian with their representative quotient, calculated using US Census Bureau data. They found that Cambodian, Filipino, Indonesian, and Laotian applicants and matriculants were underrepresented when compared to US representation.

Mitigation strategies to address underrepresentation are complex, particularly in the setting of the Supreme Court’s 2023 decision in *Students for Fair Admissions, Inc v President and Fellows of Harvard College,* which prohibits the use of race in admissions. These ethnic groups have faced additional educational, social, economic, and displacement challenges, among others, that may contribute to lower numbers seeking and attaining entry to medical school.^3^ This pattern of unequal access is not unique to the Asian experience in America.

The lack of Native American/Alaska Native students within biomedical sciences and the STEM fields is one example. Hailing from a mix of rural reservation lands and/or urban environments, possessing the aspiration and gumption to dream of being a physician, let alone learning how to navigate the circuitous route to higher education in the health sciences, can seem to be an insurmountable obstacle. However, the use of supportive research educational opportunities has been widely successful in breaking these daunting barriers, building confidence, and fostering a realization that it is okay to dream big.^4^

This approach also builds upon itself by allowing past graduates to be mentors, from senior students to early-stage investigators, further promoting self-confidence while simultaneously tapping into social homophily for all involved. When extrapolated to first-generation students, similar findings prevail for the power of supportive educational pathways.^5^ If we are stumbling and losing our way at the furtive steps on the educational journey to becoming a physician, it paints a dire portent for our ability to close the gap in physician representation.

Ethnicity and race alone are only one piece of the homophily puzzle. For example, in California, not all Filipino physicians speak Tagalog, while more physicians speak Spanish than identify as Latino/Hispanic/Spanish, and there are fewer physicians with disabilities than people living with disabilities. As Jacobs and Booth wrote, limits to federal student loans risk “creating a medical caste system in which socioeconomic status determines not only who becomes a physician but also where they practice and whom they serve”.^6^ Yang’s finding builds on work from Nguyen et al. where US medical schools saw declining entrance and acceptance rates for students who are underrepresented in medicine after the Supreme Court’s 2023 decision. As a percentage of matriculants, underrepresented students in medicine, including American Indian and Alaska Native, Black, and Hispanic students, decreased. This shift in matriculation was primarily associated with changes for schools located in states that had no prior ^7^ state-level affirmative action bans, suggesting that policies have a profound impact. And indeed, more information on the critical contributions of DOs, IMGs, and FMGs is needed.

Much of the conversation about workforce diversity centers on medical school admissions, but the training journey does not end there. Graduate medical education (GME) is where the physician workforce takes shape, where trainees refine their clinical identities, choose their subspecialties, and establish communities they will serve. Yet, the use of disaggregated data at this stage remains limited and is not routinely examined or leveraged in meaningful ways.

Examining these patterns within GME offers critical insight into who advances through training and where they will ultimately serve. Physicians from historically excluded communities are more likely to return to those communities to practice,^8,9^ and language-concordant care is linked to improved trust, adherence, and outcomes.^10^ These connections are not coincidental; they reflect how identity, language, and lived experience shape the physician-patient relationship. Precepting a Vietnamese-speaking resident in clinic as they share a cultural understanding with their patient is a powerful reminder that identity isn’t peripheral to care; it’s central to it.

If the goal is to build a physician workforce that reflects and meets the needs of an increasingly diverse US population, this lens must extend into GME. Disaggregated data can help residency programs and institutions set intentional goals: identifying gaps, aligning training with community needs, and strengthening pathways for physicians whose identities and languages mirror those of their patients. This, too, is part of honoring identity in medical education and moving beyond describing inequities toward deliberately shaping a workforce capable of advancing health equity.

Achieving a diverse physician workforce has never been easy and has never been more important. It is well worth re-examining how we ensure that the meritorious tapestry of physicians in the USA continues to meet the evolving needs of global communities.
